# Calotropis procera and the Pharmacological Properties of Its Aqueous Leaf Extract: A Review

**DOI:** 10.7759/cureus.60354

**Published:** 2024-05-15

**Authors:** Aisha Habeeb, Sindhu Ramesh, Rajeshkumar Shanmugam

**Affiliations:** 1 Conservative Dentistry and Endodontics, Sri Sai College of Dental Surgery, Hyderabad, IND; 2 Conservative Dentistry and Endodontics, Saveetha Dental College, Saveetha Institute of Medical and Technical Sciences (Deemed to be University), Chennai, IND; 3 Nanobiomedicine Lab, Centre for Global Health Research, Saveetha Medical College and Hospital, Saveetha Institute of Medical and Technical Sciences (Deemed to be University), Chennai, IND

**Keywords:** aqueous leaf extract, pharmacological activities, toxicity, chemometric profile, calotropis procera

## Abstract

*Calotropis procera (C. procera)* is a versatile plant often used for fuel, fodder, wood, fiber, phytoremediation, medicine, and synthesis of nanoparticles. Its ability to tolerate abiotic stresses and its morphophysiological adaptation have made it popular worldwide. Currently, it is identified as an environmental weed across the world. *C. procera *owes its therapeutic qualities to the secondary metabolites like tannins, alkaloids, and phenols present in it. New synthetic drugs are being formulated by using these secondary metabolites as a prototype. This review aimed to provide a summary of the chemometric profile, toxicity, and pharmacological activities of the aqueous leaf extract of *C. procera *based on the current literature.

## Introduction and background

**Figure 1:**
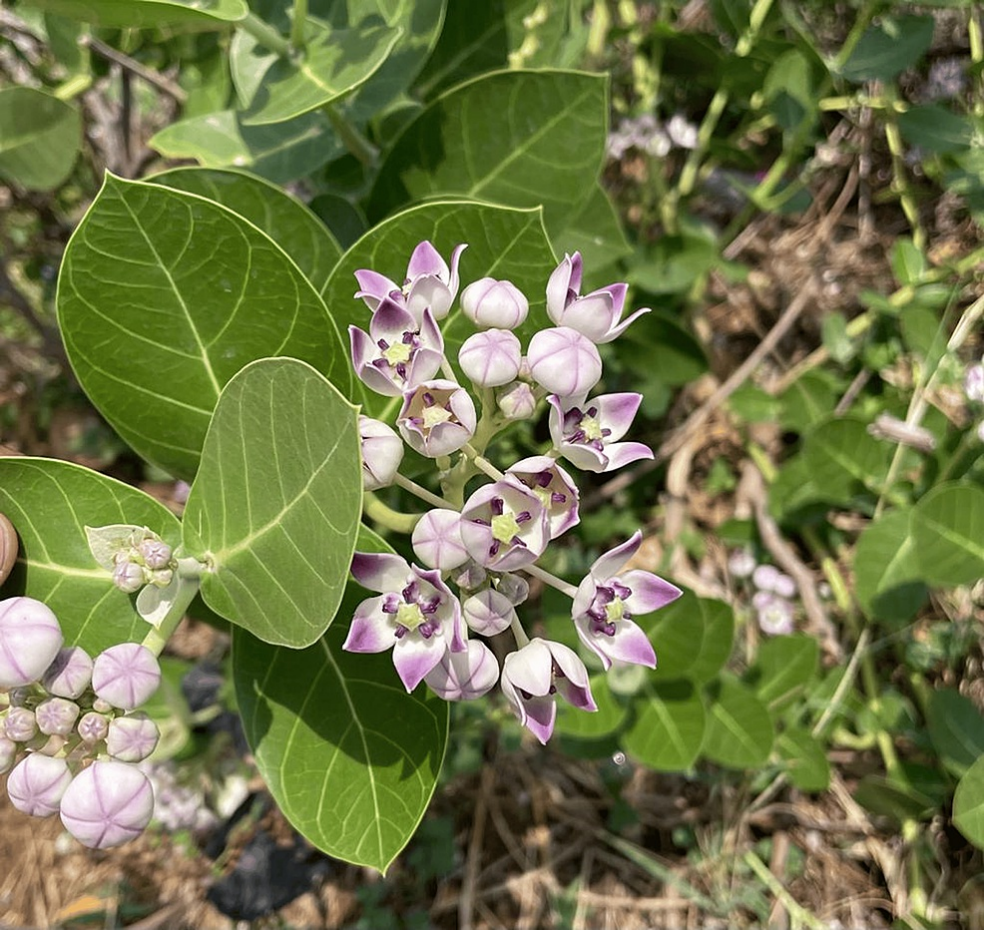
Calotropis procera

Approximately 80% of the population in the developing world uses herbal remedies for their primary healthcare needs [1]. Numerous bioactive compounds are derived from plants. One such plant that has been essentially harvested due to its characteristic medicinal properties is *Calotropis procera (C. procera) *(Figure 1). The Greek word Calotropis means beautiful (describing its flowers) and the Latin word procera refers to leaves and stem cuticular wax [2]. It is commonly known as aak, king's crown, rubber bush, sodom apple, and rubber tree and is a perennial shrub belonging to the family Apocynaceae [3,4]. It is an evergreen, softwood xerophytic plant distributed throughout the world, especially in dry and semi-arid regions of Asian and African tropical and subtropical regions [5,6].

In Ayurveda, dried leaves of *C. procera* are utilized to alleviate rheumatic pain and paralysis, and as an expectorant [7,8], and its tender leaves are used to treat migraine [9]. Moreover, its powdered leaves are used to promote faster wound healing, as a laxative, and for the treatment of indigestion [4]. Saudi Arabian traditional medicine utilizes the aerial parts of *C. procera* decoction to treat constipation, joint pain, fever, and muscular pain [10]. *C. procera* is also useful in the treatment of various skin diseases, diarrhea, and sinus fistula [11]. 

It has been reported that the solubility of bioactive compounds depends on the solvent used [12]. Aqueous extracts are generally economical, simple to prepare, and eco-friendly. Water is considered more advantageous as it is a natural solvent and has no constraints in its use. This review intends to summarize the chemometric profile, toxicity, and pharmacological activities of *C. procera* aqueous leaf extract (CALE), as available in the literature.

## Review

Preparation of CALE

It has been reported that the effectiveness of the plant extract depends on the amount of bioactive compounds dissolved in a particular type of solvent, as the degree of different phytochemical constituent dissolution differs with different solvents [13,14]. The use of water for the preparation of plant extract is comparatively more environmentally safer and water is more easily available than the majority of the organic solvents [14]. 

Various methodologies for the synthesis of CALE have been reported in the literature, which are summarized in Table 1. Initially, healthy leaves of *C. procera* are collected, washed, and shade dried. These dried leaves are later ground to obtain a fine powder. Later, a mixture of distilled water and *C. procera* leaf powder is processed to prepare CALE.

**Table 1:** CALE preparation methodology in different studies included in the present review CALE: C*alotropis procera* aqueous leaf extract

Author	Year	Location of *C. procera* plant	Preparation of CALE
Mbako et al. [15]	2009	Kaduna, Nigeria	*C. procera* fresh leaves (3.5 kg) were crushed, squeezed, and filtered. This fresh extract obtained was oven-dried into powder form at 50 °C
Nenaah and Ahmed [13]	2011	Najran City, Kingdom of Saudi Arabia	For seven days, 500 g of dried *C. procera* leaves were macerated with distilled water and the sample was intermittently shaken every two hours
Mohanraj and Usmani [16]	2012	Salem, Tamil Nadu, India	A mixture of 10 gm of dried *C. procera* leaf powder and distilled water was slowly heated for six hours and then filtered. The resulting filtrate underwent 15 minutes of centrifugation at 5000 rpm. The liquid above the sediment was collected and concentrated to one-fifth of its original volume, autoclaved, and preserved at 4 °C
Mohammed et al. [17]	2012	Jigawa State, Nigeria	For 72 h, dried *C. procera *leaf powder (50 g) was soaked in distilled water (2 L) at room temperature. The filtrate of this mixture was reduced to dark green residue using a water bath
Shobowale et al. [18]	2013	Ogun State, Nigeria.	At room temperature, 10 g dried *C. procera* leaf powder was soaked in 100 ml water for 24 h and subjected to filtration. The filtrate was evaporated with the help of a rotary evaporator at 40 °C. The resultant residue was stored at 4 °C
William et al. [19]	2015	Maiduguri, Borno State, Northeast Nigeria	To obtain a dark green CALE, a mixture of 250 g dried, pulverized *C. procera *leaves, and distilled water was refluxed for 2 hours twice. Each time fresh distilled water was used. Later, the filtrate of the mixture was concentrated at 40-50 °C using a hot air oven
Ajiboso et al. [20]	2015	Niger State, Nigeria	Filtrate of a 24-hour standing solution of 10 ml distilled water and 20g *C. procera *leaf powder was reduced to 10 ml concentrate using a water bath
Patil and Makwana [21]	2015	Nashik, India	A mixture of *C. procera* leaf powder and distilled water in a 1:16 ratio by weight was boiled to reduce its volume by 1/32 times and stored in a refrigerator
Alrheam and Shehri [22]	2015	Dawadmi, Kingdom of Saudi Arabia	At room temperature, one liter of water and 100 grams of dried *C. procera *leaf powder were soaked for four days with continuous stirring. Later, this mixture was subjected to filtration and placed in a water bath
Kazeem et al. [23]	2016	Lagos State, Nigeria	The dried *C. procera* leaf powder was immersed in water for 24 hours, following which the resultant solution underwent filtration. The solution obtained was subjected to freeze-drying, after which it was dissolved in 10% dimethyl sulfoxide (DMSO) to create a stock solution
Gawade et al. [24]	2017	Kolhapur, India	A solution containing 2 g dried crushed *C. procera* leaves and 100 ml of distilled water was boiled for 10 min, filtered, and subjected to 5 min centrifugation at 1500 rpm
Kinda et al. [25]	2019	Centre Region, Burkina Faso	At 100 ℃, a mixture of 25 g dried *C. procera* leaf powder and 500 ml distilled water was heated for 30 minutes and then filtered. At 4,000 rpm, the obtained filtrate was centrifuged for 10 minutes. The liquid portion was lyophilized and kept at 4 ℃
Rani et al. [26]	2020	Pakistan	For 24 hrs, at room temperature, a mixture of leaf powder (100 g) and distilled water (500 ml) was placed in a shaking incubator. Later, the mixture was subjected to five minutes of boiling, 15 minutes of water bath incubation at 80 °C, bench cooling, and filtration, and was stored at 4 °C
Al-Zuhairi et al. [27]	2020	Diyala Province, Iraq	A mixture of 50 g of dried *C. procera* leaf powder and 2 L of deionized water was left for 72 hours and later filtered. To obtain a dark green gummy extract, the filtrate was evaporated in a water bath
Ali et al. [28]	2020	Pakistan	At room temperature, a solution containing 100 g of *C. procera* leaf powder and 500 ml of distilled water was placed for 24 hours in a shaking incubator. The resultant solution was boiled for 5 minutes, kept in a water bath for 15 minutes at 80 °C, filtered, and stored at 4 °C
Kalu et al. [29]	2022	Nigeria	A solution containing deionized water (100 ml) and leaf powder (10 g) was maintained at a temperature of 70 °C for 1 h, cooled, filtered, and stored at 4 °C
Mamat et al. [30]	2023	Touboro, Cameroon	A macerated solution of 100 g *C. procera* leaf powder and 1000 ml distilled water was boiled for 10 minutes, filtered, and then concentrated in the oven for 72 hours at 40 °C
Nejhad et al. [31]	2023	South of Iran	For 24 hours, at ambient temperature, in a 1:10 (w/v) ratio, with stirring, *C. procera* dry leaf powder was soaked in water. The resultant solution obtained was filtered, centrifuged, concentrated, and stored at 4 °C

Chemometric profile of CALE

The medicinal properties of *C. procera* can be attributed to its phytochemical constituents. Over the years, these pharmacologically active constituents have been used in traditional medicine to treat illnesses [32]. To identify and assess the therapeutic potential of herbal extracts, it is essential to isolate and elucidate the structure of active compounds [33]. The chemometric analysis of CALE by William et al. [19] revealed the results listed in Tables 2-3. Ajiboso et al. [20] have reported the presence of 11 pharmacologically important phytochemicals, such as alkaloids, saponins, tannins, glycosides, cardenolides, steroids, anthraquinones, phenols, terpenoids, phlobatannins, and chalcones. On the contrary, Mamat et al. [30] reported the absence of anthraquinones in CALE, but an additional presence of coumarins and anthocyanins. Similarly, Kalu et al. [29] reported the presence of saponins [24.42 ± 1.96 (mg g−1)], flavonoids [33.83 ± 2.18 (mg QE g−1)], tannins [25.95 ± 1.94 (mg g−1)], phenols [43.05 ± 2.25 (mg GAE/g)], and alkaloids [8.38 ± 1.78 (mg g−1)]. Phytochemical screening of CALE conducted by Kazeem et al. [23] recorded the presence of tannins, reducing sugar, steroids, flavonoids, and saponins. Mohammed et al. [17] have documented the presence of saponins, tannins, flavonoids, alkaloids, phenolics, and terpenes in CALE.

**Table 2:** Phytochemical constituents of the aqueous leaf extract of Calotropis procera* *[15]

Phytochemical constituent	Test
Carbohydrate	General Molisch test
Flavonoids	Shinoda’s test, ferric chloride test
		Cardiac glycosides	Salkowski test, Liebermann-Burchard test
Saponin glycosides	Frothing test		
Phenols	Fluorescence analysis
Tannins	Lead acetate test, ferric chloride test

**Table 3:** Elements present in aqueous leaf extract of Calotropis procera* *[15]

Element	Concentration (%)
Lead (Pb)	0.00005
Copper (Cu)	0.000335
Nickel (Ni)	0.000027
Iron (Fe)	0.00024
Cadmium (Cd)	0.00024
Chromium (Cr)	0.000014
Zinc (Zn)	0.000022

The presence of catechin [275.2 µg/g dry weight (DW)], rutin (142.4 µg/g DW), luteolin (295.6 µg/g DW), caffeic acid (308.2 µg/g DW), kaempferol (157.9 µg/g DW), and p-coumaric acid (396.2 µg/g DW) was identified through a detailed polyphenolic content analysis of CALE conducted by Nejhad et al. They also assessed the total quantity of flavonoids, phenols, and beta-carotene of CALE to be 1781.7 ± 7.64 µg QE/g of DW, 174.82 ± 3.60 mg GAE/g of DW, and 2.26 ± 0.05 mg/100 g respectively [31]

Gas chromatography-mass spectroscopy analysis of CALE by Rani et al. [26] revealed the presence of major phytochemicals such as tridecane, tridecyl ester, mannosamine, pentatriacontane, 1-hexacosene, 1-bromo-, 2-propenoic acid, and R-limonene. The variation in the chemometric profile of CALE among studies can be attributed to the agro-environmental conditions of the places from which the plant leaves were collected and studied [22].

Toxicity

Kinda et al. [25] conducted an assessment of acute toxicity, subacute toxicity, and behavioral and psychotropic effects following the administration of CALE. The acute toxicity test performed with CALE administration at a dosage level of 3000 mg/kg body weight (b.w.) revealed zero mortality rate among mice during a 72-hour observation period. Furthermore, after two weeks of maintenance of the study animals, no change in body weight was observed, and no alterations in the macroscopic appearance of internal organs were detected during autopsy. The subacute toxicity was assessed with daily oral administration of 100, 200, and 400 mg/kg b.w. of CALE for 28 consecutive days. No mortality and no behavioral manifestations were observed throughout the 28-day monitoring duration. The body weight of the animals initially decreased slightly, but later, there was no notable variance in body weight, when comparing the findings on day 7, day 14, and day 28 to those on day 1. At all three concentrations (100, 200, and 400 mg/kg b.w. of CALE), a reduction in the creatinine concentration was observed.

An increase and decrease in alanine aminotransferase (ALT) were appreciated at the 100 mg/kg and 400 mg/kg dosage respectively. Aspartate aminotransferase (AST), total triglyceride, and urea showed no significant changes. A decrease in platelet count was observed at 100 mg/kg b.w. dosage of CALE; however, no significant change in platelet count was seen at 200 and 400 mg/kg b.w. of CALE. Four tests were conducted to determine behavioral changes in mice following the administration of 100 or 400 mg/kg b.w. CALE. The hole-board test to check the anxiety effect demonstrated a significant decrease in head dipping at the 400 mg/kg dose. The forced swimming test to check the depression effect showed no significant difference in immobility time between the tested (100 or 400 mg/kg) and control (tramadol) groups. The traction and fireplace tests were used to determine the sedative effects of CALE. The animals showed a normal response to these two tests in comparison to the diazepam control group.

Acute toxicity tests performed by Alrheam et al. on rats showed the nontoxic nature of CALE up to 2500 g/kg b.w. [22]. When orally administered at tolerable doses (80, 40, and 20 mg/kg) for 14 days, CALE showed no statistically significant change in hematological parameters (packed cell volume, hemoglobin, platelets, white blood cells, and differential leucocyte count) and biochemical parameters (serum albumin, serum protein, aspartate aminotransferase, alkaline phosphatase, and alanine aminotransferase) in female rabbits [13]. An acute toxicity study of CALE in male rabbits using the up and down method estimated the median lethal dose (LD50) to be 2435.25 mg/kg b.w. [26].

Mohammed et al. [17] conducted acute and chronic toxicity studies (60-day assessment) of CALE on Wistar albino rats. The acute toxicity study, involving oral administration of doses up to 5000 mg/kg b.w., did not result in any mortality. In chronic toxicity tests, animals showed body weight gain, with a slight increase in weight noted in the control group. Red blood cells (RBC), platelets, hemoglobin (HGB), and hematocrit (HCT) increased when doses of 500, 1000, and 1500 mg/kg b.w. were administered orally for 60 days. No significant changes in white blood cell count, mean corpuscular hemoglobin, mean corpuscular hemoglobin concentration, monocytes, granulocytes, mean corpuscular volume, lymphocytes, and serum markers for kidney function (urea and creatinine) and liver function (AST and ALT) were detected in comparison to the control group. At a dosage of 1500 mg/kg body weight, mild necrosis and vascular degenerative changes were detected.

The MTT assay, conducted by Nejhad et al. [31] using the HT-29 (human colorectal adenocarcinoma cell line) against CALE, described it as dose-dependent, with an estimated IC50 (half-maximal inhibitory concentration) value of 236.87 ± 1.46 μg/mL.

Potential biomedical applications

Synthesis of Nanoparticles (NPs)

Plant extract-aided green synthesis of NPs is eco-friendly, economical, and less time-consuming [34]. Highly stable NPs are produced when individual phytochemicals are used, and the quality of NPs is influenced by the hydroxyl groups of these phytochemicals [34]. Ali et al. [27] synthesized iron oxide NPs using CALE. These NPs (1 mg/ml) showed antifungal properties comparable to chemical fungicides (metalaxyl + mancozeb). CALE facilitated the synthesis of magnetite (Fe3O4) NPs of size 62.83 nm [0.241 polydispersity index (PDI)] when a phenolic constituent of CALE was used as capping agent, 68.02 nm (0.186 PDI) when flavonoid constituent of CALE was used as capping agent, and 134 nm (0.323 PDI) when saponin constituent of CALE was used as capping agent. The flavonoid constituent of CALE resulted in larger but stable magnetite NPs. The ascending order of antimicrobial effectiveness for magnetite nanoparticles was as follows: *Escherichia coli*, *Klebsiella pneumonia*, *Fusarium oxysporum*, *Aspergillus niger*, *Bacillus subtilis*, and *Staphylococcus aureus* [29]. Gawade et al. [24] have reported that Zinc oxide nanoparticles (ZnONPs) synthesized using CALE were spherical in shape with 15 to 24 nm in size. FT-IR analysis indicated the presence of aldehydes, amines, hydroxyl groups, carboxylic acids, and ketones in CALE, which might be responsible for the biochemical synthesis of ZnONPs [24].

Antimicrobial Property

The bioactive constituents of CALE resulted in the inhibition of microorganisms, which increased with a rise in temperature, as shown in Table 4 [18].

**Table 4:** Antimicrobial activity of the aqueous leaf extract of Calotropis procera*

Study	Microorganism	Zone of inhibition (mm)
Shobowale et al. [18]	Escherichia coli	10
	Salmonella typhi	10
	Bacillus subtilis	0.0
	Candida albicans	10
	Aspergillus niger	10

Antimicrobial Activity

The growth of *Escherichia coli*, *Aspergillus niger*, *Salmonella typhi*, and *Candida albicans *was inhibited by CALE with minimum inhibitory concentration (MIC), as represented in Table 5 [18]. In contrast, Nenaah et al. [13] reported a weak antimicrobial activity of CALE against *Escherichia coli* and *Staphylococcus epidermidis* with 8.0 ±0.05 mm and 7.5 ±0.25 mm zones of inhibition respectively. The antifungal activity of CALE was demonstrated, showing the largest zone of inhibition against *Candida tropicalis* (14.5 ±0.80 mm), followed by *Penicillium chrysogenum* (12.5 ±0.65 mm), *Saccharomyces cerevisiae *(12.0 ±0.45 mm), *Candida albicans* (11.5 ±0.60 mm), *Aspergillus flavus *(11.0 ±0.10 mm), and lastly *Aspergillus niger*(10.0 ±0.40 mm) [13].

**Table 5:** Minimum inhibitory concentration of CALE CALE: *Calotropis procera* aqueous leaf extract; MIC: minimum inhibitory concentration; N: not determined

Study	Bacteria	MIC value (mg/ml)
Shobowale et al. [18]	Escherichia coli	10
	Salmonella typhi	10.5
	Bacillus subtilis	N
	Candida albicans	N
	Aspergillus niger	15

Antidiabetic Potential

In 2019, 463 million people were estimated to be diabetic globally. The diabetic population is projected to rise to 578 million by 2030 and reach 700 million by 2045 [35]. Hyperglycemia is defined as a state when blood glucose increases rapidly due to the action of pancreatic α-amylase in breaking down starch and the subsequent action of intestinal α-glucosidases in absorbing glucose [36]. Thus, suppressing these carbohydrate hydrolyzing enzymes helps in reducing postprandial hyperglycemia and offers a crucial approach to managing diabetes mellitus [37]. α-amylase at a concentration of 15.75 ± 1.05 mg/mL and α-glucosidase at a concentration of 3.25 mg/mL were strongly inhibited by CALE under in-vitro conditions [22].

*Anti-hyperbilirubinemic*****

Phenylhydrazine and paracetamol-induced hyperbilirubinemic Wistar rat models treated with CALE have shown a reduction in the total serum bilirubin levels, similar to silymarin, which has documented hepatoprotective properties. The anti-hyperbilirubinemic property of CALE can be attributed to its bilirubin-lowering property exhibited by antioxidant phytochemicals, thus resulting in hepatocyte plasma membrane stabilization [21].

Wound-Healing Activity

Wistar rat models treated with CALE at a dose of 25 mg/kg for incision wounds and 50 mg/kg for excision wounds showed a significant increase in the breaking strength of sutured skin and a greater wound contraction rate, leading to a reduction in the epithelization period, respectively. The wound-healing property of CALE could be due to chemical moieties leading to increased fibrinogenesis [21].

Antioxidant Potential

It is well-known that oxidative damage to DNA is directly related to the development of cancer [38]. Various studies have linked oxidative stress to several cardiovascular diseases such as congestive heart failure, cardiomyopathy, atherosclerosis, hypertension, cardiac hypertrophy, and ischemia [39-43]. It has been reported that oxidative stress results in the production of toxic peptide ß-amyloid, which plays a crucial role in degenerative neurological conditions [44]. Oxidative stress even enhances the inflammatory process by increasing NF-kappa B and AP-1 levels, thus complicating pulmonary inflammatory disorders [45]. Oxidative stress is also involved in the development of rheumatoid arthritis due to the generation of reactive nitrogen and oxygen free radicals in and around the joints [46]. Renal diseases such as chronic renal failure, glomerulonephritis and tubulointerstitial, uremia, nephritis, proteinuria, and cataracts along with age-related macular degeneration are all induced by oxidative stresses [47,48]. Antioxidants play an important role in the neutralization of these oxidative stresses, thus preventing various diseases [49].

Plants are a rich source of antioxidants. The antioxidant properties of a plant are directly proportional to its antioxidant content, especially the phenolic content and concentration of the extract [50]. The phytochemical analysis of CALE has reported the presence of phenols and beta-carotene [15-17,20]. Beta-carotene is a strong antioxidant and the most effective suppressor of singlet oxygen [31]. Free radical DPPH (1, 1-diphenyl-2-picryl-hydrazyl) scavenging ability, nitric oxide radical inhibition, and reducing power test have revealed the antioxidant potential of CALE, which was dose-dependent [16]. Similar dose-dependent antioxidant activity of CALE was observed in the study performed by Nejhad et al. using the ferric reducing antioxidant power (FRAP) assay, ABTS (2,2'-Azino-bis(3-ethylbenzothiazoline-6-sulfonic acid)) free radical cation scavenging assay, and free radical DPPH scavenging assay. However, both the natural antioxidant (vitamin C) and the synthetic antioxidant [tertiary butylhydroquinone (TBHQ)], which were used as controls, were found to be twice as effective as CALE [31].

Hematopoietic Effect

In Burkina Faso, a combination of *C. procera *and *Zanthoxylum zanthozyloïdes* plants are used for the treatment of sickle cell disease [51]. The hematopoietic effect of CALE has been described, with a significant increase in HGB, HCT, and RBC levels after 60 days of administration at doses of 500, 1000, and 1500 mg/kg b.w. [17]. This is particularly important for treating anemic conditions.

## Conclusions

The aqueous leaf extract of *C. procera*, an evergreen plant growing in barren lands with a worldwide distribution, has been shown to contain beneficial phytochemicals. It has been demonstrated that CALE has antimicrobial activity and antidiabetic potential, as well as anti-hyperbilirubinemic and wound-healing abilities. Further research is required to effectively employ the synergistic effects of these phytochemicals commercially, which could have a remarkable impact as we are constantly combating the rising incidence of superbugs, diabetes, hyperbilirubinemia, and failure of post-surgical wound healing.
